# A Tale of Two Biomarkers: Untargeted ^1^H NMR Metabolomic Fingerprinting of BHBA and NEFA in Early Lactation Dairy Cows

**DOI:** 10.3390/metabo10060247

**Published:** 2020-06-15

**Authors:** Timothy D. W. Luke, Jennie E. Pryce, William J. Wales, Simone J. Rochfort

**Affiliations:** 1 Agriculture Victoria Research, AgriBio, Centre for AgriBioscience, Bundoora, VIC 3083, Australia; tim.luke@agriculture.vic.gov.au (T.D.W.L.); jennie.pryce@agriculture.vic.gov.au (J.E.P.); 2 School of Applied Systems Biology, La Trobe University, Bundoora, VIC 3083, Australia; 3 Agriculture Victoria Research, Ellinbank Centre, Ellinbank, VIC 3821, Australia; bill.wales@agriculture.vic.gov.au; 4 Centre for Agricultural Innovation, School of Agriculture and Food, Faculty of Veterinary and Agricultural Sciences, The University of Melbourne, Parkville, VIC 3010, Australia

**Keywords:** metabolic profile, ketosis, transition period, livestock, methyl donor, one-carbon metabolism, negative energy balance

## Abstract

Disorders of energy metabolism, which can result from a failure to adapt to the period of negative energy balance immediately after calving, have significant negative effects on the health, welfare and profitability of dairy cows. The most common biomarkers of energy balance in dairy cows are β-hydroxybutyrate (BHBA) and non-esterified fatty acids (NEFA). While elevated concentrations of these biomarkers are associated with similar negative health and production outcomes, the phenotypic and genetic correlations between them are weak. In this study, we used an untargeted ^1^H NMR metabolomics approach to investigate the serum metabolomic fingerprints of BHBA and NEFA. Serum samples were collected from 298 cows in early lactation (calibration dataset *N* = 248, validation *N* = 50). Metabolomic fingerprinting was done by regressing ^1^H NMR spectra against BHBA and NEFA concentrations (determined using colorimetric assays) using orthogonal partial least squares regression. Prediction accuracies were high for BHBA models, and moderately high for NEFA models (R^2^ of external validation of 0.88 and 0.75, respectively). We identified 16 metabolites that were significantly (variable importance of projection score > 1) correlated with the concentration of one or both biomarkers. These metabolites were primarily intermediates of energy, phospholipid, and/or methyl donor metabolism. Of the significant metabolites identified; (1) two (acetate and creatine) were positively correlated with BHBA but negatively correlated with NEFA, (2) nine had similar associations with both BHBA and NEFA, (3) two were correlated with only BHBA concentration, and (4) three were only correlated with NEFA concentration. Overall, our results suggest that BHBA and NEFA are indicative of similar metabolic states in clinically healthy animals, but that several significant metabolic differences exist that help to explain the weak correlations between them. We also identified several metabolites that may be useful intermediate phenotypes in genomic selection for improved metabolic health.

## 1. Introduction

Most dairy cows experience a period of negative energy balance immediately after calving due to both a reduction in feed intake preceding calving [1], and an increase in energy requirements for milk production [2]. A successful transition from pregnancy to lactation requires a series of complex and coordinated changes in metabolism and nutrient partitioning, known as homeorhesis [3]. Failure of these homeorhetic controls can lead to the development of metabolic disorders such as ketosis and fatty liver [4]. These disorders can have significant negative effects on the health, welfare and profitability of early-lactation dairy cows due to their (1) relatively high incidence [5,6], (2) demonstrated association with other diseases [4,7] and (3) their significant economic costs [8,9].

Serum β-hydroxybutyrate (BHBA) and non-esterified fatty acids (NEFA) are biomarkers that are commonly used to evaluate the energy balance of dairy cows in the transition period [6,10,11]. One of the main physiological responses to reduced energy intake is the mobilization of stored energy from adipose tissue as NEFA. Serum NEFA concentration is a measure of the degree of lipolysis, and therefore an indicator of the magnitude of negative energy balance [12]. Once released, NEFA are transported via the bloodstream to the mammary gland for milk fat synthesis, or to the liver where they undergo either (1) complete oxidation via the TCA cycle, (2) partial oxidation to ketone bodies (BHBA, acetone and acetoacetate), or (3) re-esterification to form triglycerides which can either be stored or exported as very low density lipoprotein (VLDL). BHBA is the most stable of the three ketone bodies [13], and is commonly used as a biomarker of energy balance [14]. 

Mild elevations in serum BHBA and/or NEFA concentration during the transition period are considered normal [15], but marked elevations are indicative of excessive negative energy balance and/or perturbed metabolism [16]. Elevated concentrations of both BHBA and NEFA can be observed in clinically healthy animals (i.e., showing no visible signs of illness), and are associated with (1) reduced reproductive performance [11,17], (2) an increased incidence of clinical diseases such as displaced abomasa and metritis [15,17,18], (3) decreased milk production [6,11,19] and (4) an increased risk of culling [6,15,20]. However, despite these similarities, both the phenotypic [21,22] and genetic [23] correlations between these two biomarkers are low. This is not necessarily important if biomarkers are being used for management purposes (such as the identification of sick animals or the assessment of nutritional status) but may be significant if the biomarkers are used as phenotypes for genetic selection for improved animal health and resilience. There is therefore a need to better understand the metabolic states represented by BHBA and NEFA.

Untargeted metabolomics combines high throughput molecular analytical techniques such as proton nuclear magnetic resonance (^1^H NMR) spectroscopy with multivariate statistical modelling, to characterize the metabolic response of a biological system to pathophysiological stimuli [24]. Examples in dairy cattle include studies of ketosis [25,26], fatty liver [27], hypocalcaemia [28] and displaced abomasa [29]. The collective metabolic features of a given state or condition can be described as its “metabolomic fingerprint”. As well improving our understanding of the biological processes, metabolomic studies can uncover intermediate molecular phenotypes (metabotypes) associated with complex animal health traits such as metabolic resilience. These metabotypes can then be integrated with genomic data to (1) elucidate the genetic architecture of these traits, and (2) improve genomic prediction accuracies [30,31].

The aim of this study was therefore to use an untargeted ^1^H NMR metabolomic approach to investigate the metabolomic fingerprints of serum BHBA and NEFA concentrations in clinical healthy dairy cows in early lactation, and in so doing (1) identify common and differential metabolic pathways, and (2) identify novel metabotypes for application to genetic selection for improved metabolic health.

## 2. Results

### 2.1. Analysis of Experimental Metadata

Descriptive statistics of the datasets used in this experiment are shown in Table 1. BHBA concentrations were significantly higher in Dataset 1 than in Dataset 2 (*p* < 0.001). The differences in all other parameters were not statistically significant (*p* > 0.05). The correlation between BHBA and NEFA concentrations was 0.45 in Dataset 1 and 0.40 in Dataset 2. 

### 2.2. ^1^H NMR Spectra

**Figure 1:**
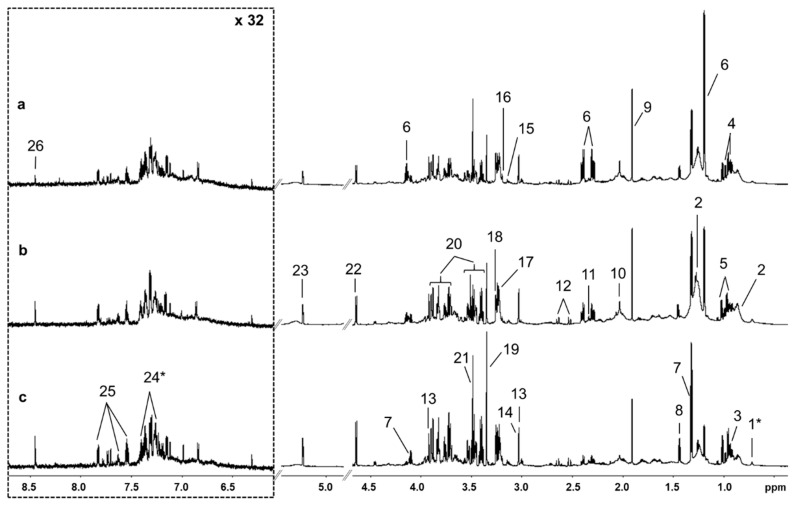
Representative 700 MHz ^1^H nuclear magnetic resonance spectra of serum samples from early lactation dairy cows with (**a**) elevated β-hydroxybutyrate (BHBA), (**b**) elevated non-esterified fatty acid (NEFA), and (**c**) normal BHBA and NEFA concentrations. Downfield regions were vertically expanded 32 times for clarity. Legend: 1, cholate; 2, very low density lipoprotein/low density lipoprotein; 3, leucine; 4, isoleucine; 5, valine; 6, β-hydroxybutyrate; 7, lactate; 8, alanine; 9, acetate; 10, N-acetyl glycoprotein; 11, pyruvate; 12, citrate; 13, creatine; 14, creatine phosphate; 15, dimethyl sulfone (DMSO_2_); 16, choline; 17, phosphocholine; 18, betaine; 19, methanol; 20, glucose; 21, glycine; 22, β-Glu; 23, α-Glu; 24, 3-phenyllactate; 25, hippurate; 26; formate. * = tentative identification.

Twenty-four metabolites could be clearly identified from the ^1^H NMR spectra. Two metabolites, cholate and 3-phenyllactate, were tentatively identified. Figure 1 shows representative spectra from animals in Dataset 1 with (a) elevated BHBA concentration, (b) elevated NEFA concentration and (c) normal BHBA and NEFA concentrations. Upfield regions of spectra were dominated by branched-chain amino acids (leucine, isoleucine and valine), organics acids (BHBA, lactate, acetate) and the methyl and methylene groups of low density (LDL) and very low density lipoproteins (VLDL) at δ 0.86 ppm and δ 1.25 ppm, respectively [32]. We also observed a prominent peak at δ 2.03 ppm which was consistent with the N-acetyl groups of glycoproteins [33]. The singlet at δ 3.14 ppm was identified as dimethyl sulfone (DMSO_2_) [34,35]. The middle of the spectrum was complex and dominated by glucose. Signal overlap and weak 2D signal strength meant that hippurate was the only compound that could be clearly identified in the downfield region. Relative chemical shifts and the multiplicity of identified peaks are available in the supplementary material (Table S1).

Unsupervised analysis of the data using PCA showed no obvious clustering of samples by dataset. Results of ANOVA-simultaneous component analysis showed that fixed effects (cow age, herd of origin and days in milk (DIM)) explained only 13.94% of the spectral variation (Table S2). Only the effect of age was statistically significant (*p* < 0.05). This suggests that most spectral variation is due to differences between individual animals. 

### 2.3. Accuracy and Robustness of Prediction Models

**Figure 2:**
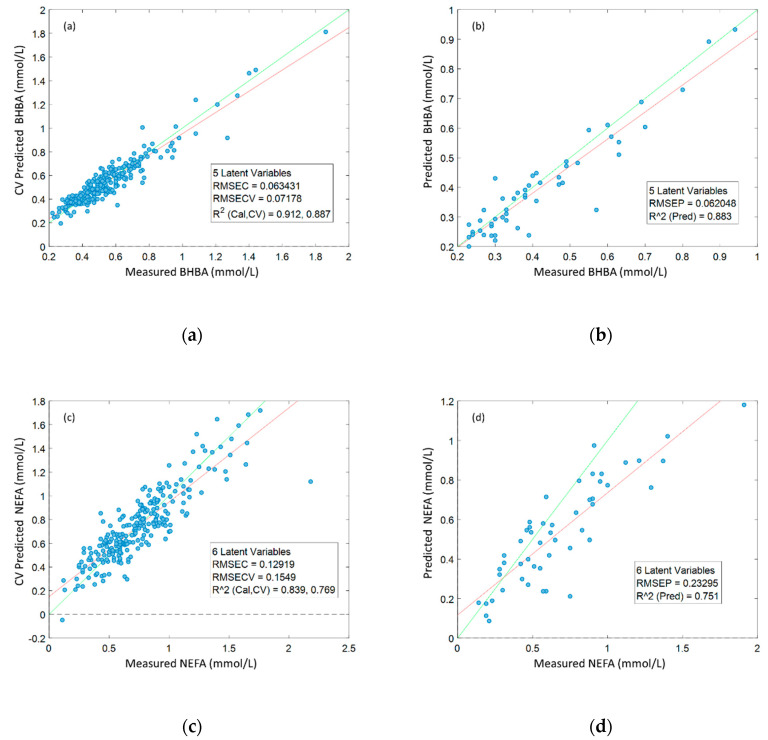
Accuracy of orthogonal partial least squares (OPLS) regression models predicting serum β-hydroxybutyrate (BHBA) and non-esterified fatty acid (NEFA) concentrations from ^1^H NMR spectra, built using data from Dataset 1 (*N* = 248); (**a**) 10-fold cross-validation (CV)-predicted BHBA vs. measured BHBA; (**b**) external validation (*N* = 50)-predicted BHBA vs. actual BHBA; (**c**) CV-predicted NEFA vs. measured NEFA; (**d**) external validation-predicted NEFA vs measured NEFA.

The robustness of the orthogonal partial least squares (OPLS) regression models built using data from Dataset 1 was assessed using (1) 10-fold cross-validation (Figure 2a,c) and (2) external validation with data from Dataset 2 (Figure 2b,d). Prediction accuracies derived from external validation were high for BHBA (R^2^ = 0.88), and moderately high for NEFA (R^2^ = 0.75). BHBA models were remarkably robust, with external validation R^2^ and RMSE results almost identical to cross-validation results. Models predicting serum NEFA concentration were less accurate than those predicting BHBA (NRMSE 0.32 and 0.50, respectively), but external validation results indicated that these models were still quite robust. *p*-values derived from permutation testing were < 0.001 for all models, indicating that models were not over-fitted.

### 2.4. Metabolomic Fingerprints of BHBA and NEFA

**Figure 3:**
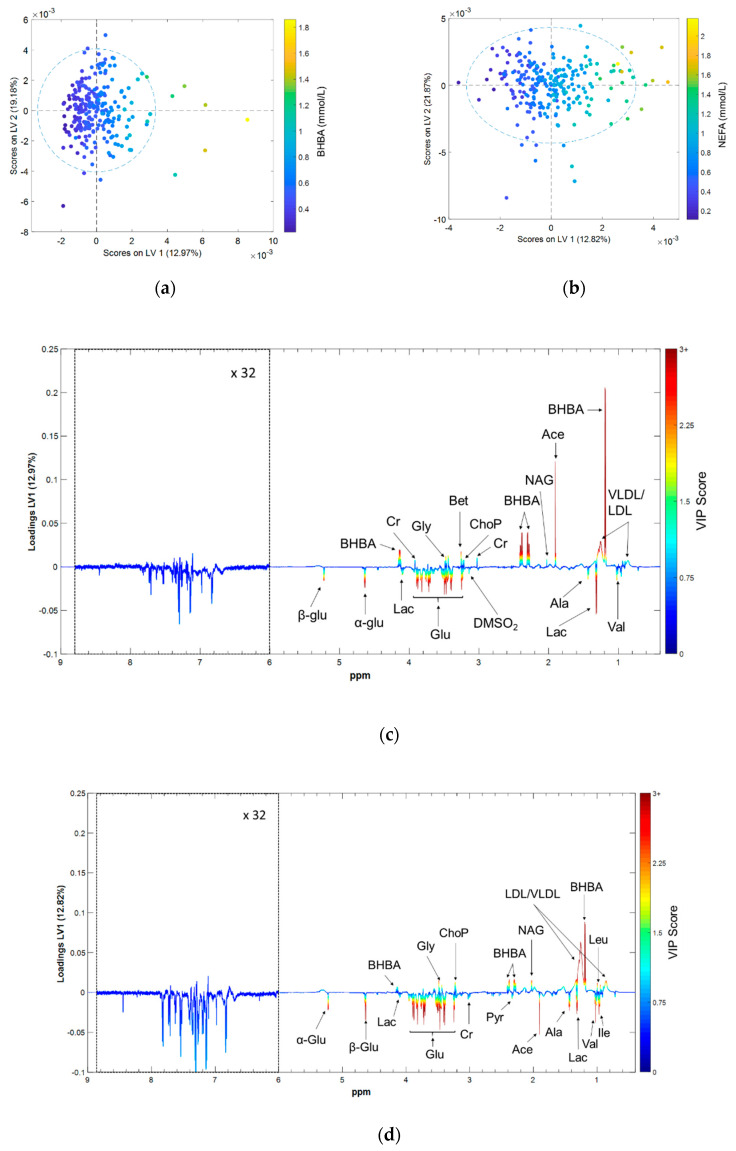
Results of the orthogonal partial least squares (OPLS) regression models predicting serum BHBA and NEFA concentrations from ^1^H NMR spectra; (**a**) First latent variable (LV1) vs. second latent variable (LV2) scores for the BHBA prediction model; (**b**) LV1 vs. LV2 scores for the NEFA prediction model; (**c**) LV1 loadings for the BHBA prediction model; (**d**) LV1 loadings for the NEFA prediction model. Scores plots color-coded by reference biomarker concentration, loadings plots by VIP score. α-Glu = α glucose, β-Glu = β glucose, Ace = acetate, Ala = alanine, Bet = betaine, BHBA = β hydroxybutyrate, Cr = creatine, DMSO_2_ = dimethyl sulfone, Glu = glucose, Gly = glycine, Ile = isoleucine, Lac = lactate, Leu = leucine, NAG = N-acetyl glycoprotein, ChoP = phosphocholine, Pyr = pyruvate, Val = valine, LDL = low density lipoprotein; VLDL = very low density lipoprotein.

The metabolomic fingerprints associated with BHBA and NEFA were investigated using OPLS regression. Larger scores on the first latent variable (LV1) correspond to higher concentrations of both BHBA and NEFA (Figure 3a,b). LV1 loadings plots were used to identify which spectral features contributed most to the variation in the reference biomarker concentrations [36] (Figure 3c,d). Spectral features with positive loadings correspond to metabolites that are positively correlated with reference biomarker concentrations, and vice-versa. Peaks with a variable importance of projection (VIP) score greater than one were considered statistically significant [37] (Figure S2).

#### 2.4.1. Commonalities in the Metabolomic Fingerprints of BHBA and NEFA

The results of this study show that several metabolites showed similar co-variances with both BHBA and NEFA concentrations. The largest effect we observed was from peaks assigned to glucose, which were negatively correlated with both biomarkers. Other metabolites with common co-variances included lactate, valine and alanine (negatively correlated), and glycine and phosphocholine (positively correlated). Spectral regions attributed to lipoproteins (LDL and VLDL) and glycoproteins were positively correlated with both BHBA and NEFA concentrations.

#### 2.4.2. Differences between the Metabolomic Fingerprints of BHBA and NEFA

**Figure 4:**
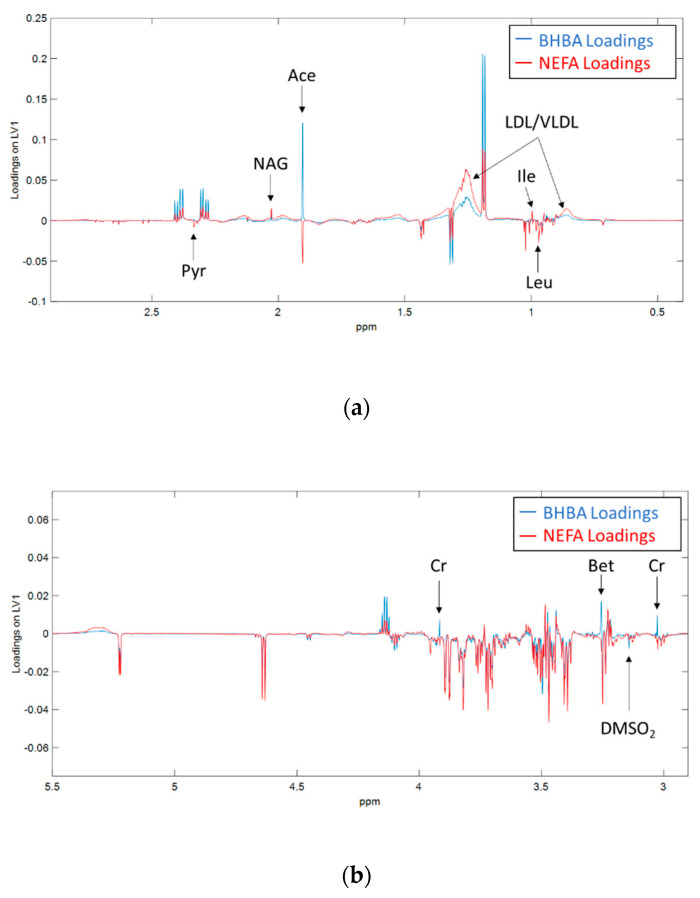
Loadings on the first latent variable (LV1) derived from orthogonal partial least squares (OPLS) regression of ^1^H NMR spectra against serum BHBA (blue) and NEFA (red) concentrations in early lactation dairy cows. Spectral regions between (**a**) δ 0.2 ppm to 2.9 ppm and (b) δ 2.9 ppm to 5.5 ppm are shown. Figure (**b**) has been for clarity purposes. Ace = acetate, Bet = betaine, ChoP = Phosphocholine, Cr = creatine, DMSO_2_ = dimethyl sulfone, Ile = isoleucine, Leu = leucine, LDL/VLDL = low/very low-density lipoprotein, NAG = N-acetyl glycoprotein, Pyr = pyruvate.

Figure 4 highlights the differences we observed between the metabolomic fingerprints of BHBA and NEFA. Acetate and creatine were positively correlated with BHBA, and negatively correlated with NEFA. A small number of metabolites showed significant co-variance with only one of the biomarkers. BHBA concentration was positively correlated with betaine, and negatively correlated with dimethyl sulfone (DMSO_2_), while NEFA concentration was positively correlated with isoleucine and negatively correlated with leucine.

## 3. Discussion 

### 3.1. Similarities between BHBA and NEFA

**Figure 5:**
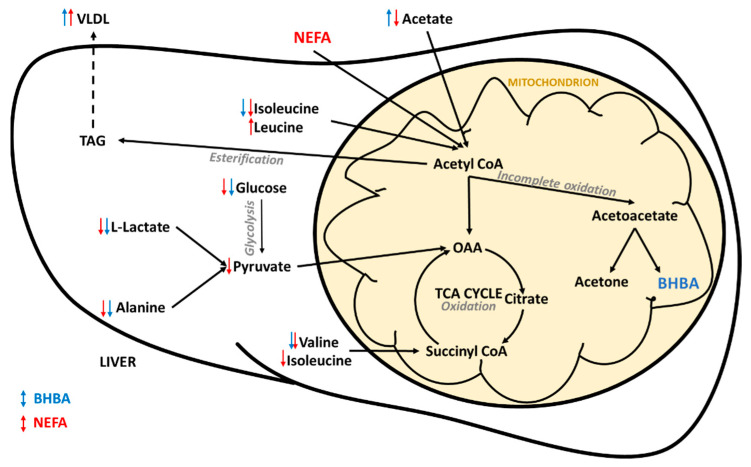
Summary of hepatic energy metabolism in early lactation dairy cows. Arrows indicate the direction of the relationship between the metabolites and the reference BHBA (blue) and non-esterified fatty acid (NEFA) (red) concentrations. BHBA = β-hydroxybutyrate; OAA = oxaloacetate; TAG = triglyceride, TCA = tricarboxylic acid, VLDL = very low density lipoprotein.

Not surprisingly, many of the metabolites identified as having common co-variance with both BHBA and NEFA concentrations are involved in hepatic energy metabolism. These relationships are summarized in Figure 5. Most obvious was the negative relationship between both biomarkers and glucose. Hypoglycaemia has been widely reported in early lactation dairy cows due to the massive demand for glucose for lactogenesis [3,38]. More recently, NMR metabolomics studies have identified serum glucose concentration as being (1) directly correlated to energy balance (r = 0.84) [39], and (2) lower in cows with clinical and subclinical ketosis [25] and fatty liver [27] when compared to healthy controls. Our results offer further evidence of the pivotal role glucose plays in the early lactation metabolic health in dairy cows.

Lactate and alanine, important gluconeogenic substrates in ruminants [40,41], were also negatively associated with both BHBA and NEFA, as was valine (another gluconeogenic amino acid). Interestingly, Xu et al. [39] found no correlation between calculated energy balance in early lactation dairy cows and the concentrations of any of the branched-chain amino acids or lactate. Conversely, when compared to healthy controls, cows with fatty liver and displaced abomasa have been shown to have lower serum alanine concentrations [27,29], and cows with ketosis have lower lactate and alanine concentrations [25,42]. This suggests that alterations in glucogenic precursors, in particular lactate and alanine, are indicative of a perturbed metabolism, not simply negative energy balance. We previously showed that lactate concentration in pasture-fed dairy cows is heavily influenced by herd-specific management factors [43], and as such may not be heavily influenced by genetic factors. Alanine has been shown to be the most important glucogenic amino acid, and the most important gluconeogenic precursor after lactate and propionate, in dairy cows [41]. Therefore, genetic selection for cows with higher serum concentrations of alanine in early lactation may help to increase endogenous glucose supply.

Spectral features attributed to VLDL and LDL were positively correlated with the concentrations of both BHBA and NEFA. These results need to be interpreted with caution as the methanol extraction used in this study removed much of the protein from the samples and may have introduced experimental artefacts. Interestingly, ^1^H NMR spectroscopy has recently been shown capable of providing high-throughput and accurate quantification of lipoprotein subclasses in human serum and plasma samples [32,44]. It is important to note that these protocols used different pulse sequences and involved the dilution of plasma/serum in a deuterated water/phosphate buffer solution without any metabolite extraction, such as the one used in our study. The findings of these studies cannot, therefore, be applied directly to our results. However, lipoprotein metabolism is central to early lactation health in dairy cows, and impaired VLDL production in the liver can result in hepatic triglyceride (TAG) accumulation (Figure 4) and the development of fatty liver [45]. Dyslipoproteinaemia is also an important feature of metabolic syndrome in humans, and the quantification of lipoprotein subclasses is considered critical to the better understanding of this disease [44]. We believe that the investigation of serum lipoproteins using ^1^H NMR spectroscopy holds great promise in the research of early lactation metabolic health in dairy cows, and we plan to validate the aforementioned protocols on bovine serum and plasma samples. 

The region of the spectrum associated with glycoproteins was also significantly positively correlated with both NEFA and BHBA concentrations. Glycoproteins are acute phase proteins which can be used as indicators of inflammation in cattle [46]. In dairy cattle, increased serum NEFA concentrations in early lactation are associated with uncontrolled inflammation, and this inflammatory dysfunction is hypothesized to be a central link between metabolic and infectious disorders [14,47]. ^1^H NMR spectroscopy is showing promise for the quantification of glycoprotein A (GlcA) in human research into metabolic diseases such obesity, diabetes mellitus and the metabolic syndrome [33]. Given that these syndromes have much in common with early lactation metabolic disease in dairy cows (e.g., insulin resistance), we believe that further research into GlcA as a biomarker for early lactation health is warranted. Overall, our results offer further evidence that inflammation plays an important role in early lactation metabolic health of dairy cows. 

Glycine was positively correlated with the concentrations of both BHBA and NEFA. Metabolomics studies comparing healthy and ketotic dairy cows have reported (1) no change in glycine concentrations [25], (2) increased glycine concentrations in cows with sub-clinical ketosis [26], (3) increased glycine concentrations in cows with clinical ketosis [48] and (4) decreased glycine concentrations in cows with clinical ketosis [26] and fatty liver [49]. Glycine concentration has also been shown to increase in response to lipolysis [50]. These differing results suggest that changes in glycine concentration may be dependent on the severity of the metabolic disorder (i.e., increased in mild cases, and decreased in more severe cases). Most interesting are the findings of a recent metabolomics study that showed that glycine concentrations in plasma and milk were strongly negatively correlated with energy balance in early lactation dairy cows (r = −0.80 and r = −0.79, respectively) [39]. The authors of this study hypothesized that this relationship was due to an increase in one-carbon or methyl donor metabolism, specifically an increase in the conversion of choline to glycine. Given that all cows in our study were clinically healthy, our results are consistent with glycine being an indicator of negative energy balance, lipolysis, and/or sub-clinical ketosis. Further work is required to better understand the role of glycine metabolism in clinical metabolic disease.

**Figure 6:**
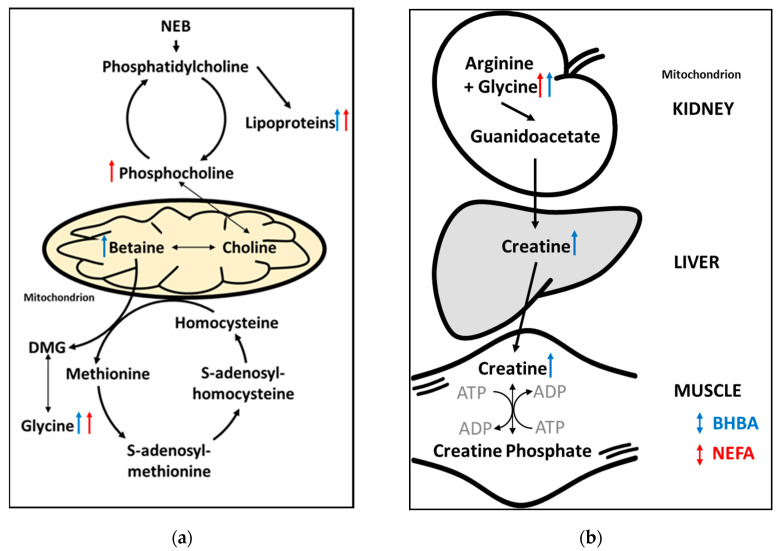
Summary of (**a**) phospholipid and one-carbon/methyl donor metabolism [53,54], and (**b**) creatine metabolism in early lactation dairy cows. Arrows indicate the direction of the relationship between the metabolites identified using untargeted ^1^H NMR metabolomics, and reference BHBA (blue) and non-esterified fatty acids (NEFA) (red) concentrations. ADP = adenosine diphosphate; ATP = adenosine triphosphate; DMG = dimethylglycine; NEB = negative energy balance.

The positive correlations between phosphocholine and both BHBA and NEFA concentrations, and between betaine and BHBA concentration, are consistent with an increase in methyl donor metabolism in cows experiencing negative energy balance. Methyl donor metabolism and nutrition are receiving a great deal of attention in dairy science due to links with early-lactation cow health (including fatty liver), milk production and immune function [51]. Betaine, phosphocholine and glycine are intermediates in several important one-carbon metabolic pathways including the folate and methionine cycles, and the cytidine diphosphate (CDP)–choline pathway [51] (Figure 6a). The positive correlation between NEFA and phosphocholine may be due to increased breakdown of phosphatidylcholine (Figure 6a). This is consistent with the findings of Imhasly et al. [52] who showed that serum concentrations of lyso-phosphatidylcholines and phosphatidylcholines increase in response to negative energy balance in post-partum dairy cows. The positive association observed between betaine and BHBA could be due to increased oxidation of choline. A detailed description of these pathways is beyond the scope of this study, however our results suggest that methyl donor metabolism has an important influence on both BHBA and NEFA concentrations in early-lactation dairy cows. 

### 3.2. Differences between BHBA and NEFA

Despite many similarities, we observed some significant differences between the metabolomic fingerprints of BHBA and NEFA. Most obvious was the difference in the direction of correlation between acetate and the two biomarkers. Acetate is a volatile fatty acid produced by microbial fermentation of feedstuffs in the rumen, and is an important energy source [55] (via oxidation or the partial oxidation of acetyl-CoA in the liver) and substrate for de novo milk fat synthesis [56] in cows. The negative relationship we observed between acetate and NEFA is consistent with the findings of Bielak et al. [57], who reported a negative correlation (r = 0.44) between plasma NEFA and acetate concentrations in early lactation dairy cows, possibly due to the down-regulation of the active transport of acetate across the rumen wall. The positive association between acetate and BHBA is consistent with previously discussed metabolomic studies of ketosis and fatty liver [25,27]. These results suggest that differences in acetate metabolism may help to explain the weak correlation between serum BHBA and NEFA concentrations in early lactation dairy cows.

The positive correlation between creatine and BHBA concentration is consistent with previous reports that creatine is a potentially useful biomarker of ketosis and severe energy deficiency in dairy cows [25,26,39]. Creatine is an important intermediate in energy metabolism, and this result may represent increased breakdown of creatine phosphate in skeletal muscle and the release of high-energy phosphate for the conversion of adenosine diphosphate (ADP) to adenosine triphosphate (ATP) (Figure 6b). Interestingly, creatine concentration was negatively correlated with NEFA concentration (albeit weakly and non-significantly (VIP < 1)). That mobilization of energy from skeletal muscle is a feature of the BHBA metabolomic fingerprint, but not that of NEFA, suggests that elevated BHBA concentrations are indicative of a more severe energy deficiency than are elevated NEFA concentrations. However, the ability to rapidly mobilize energy from skeletal muscle may be advantageous to early-lactation dairy cows, and we believe the role of creatine metabolism in transition cow health warrants further investigation. We therefore plan to undertake genome-wide association studies to better understand the genetic relationships between hepatic and skeletal muscle energy metabolism.

The significant negative correlation between DMSO_2_ and BHBA concentration was an interesting finding of this study. DMSO_2_ concentration in the milk and rumen fluid of dairy cows has been shown to vary according to feeding system; higher in pasture-fed cows managed outdoors than in cows fed a total mixed ration indoors [58]. Maher et al. [59] showed that the concentrations of DMSO_2_ in milk and plasma are highly correlated (r = 0.69), so serum DMSO_2_ may also be an indicator of pasture intake. Given that all animals in this experiment were fed pasture, the negative association we observed between DMSO_2_ and BHBA concentration may indicate that hyperketonemic cows are consuming less feed.

## 4. Materials and Methods 

All procedures undertaken in this study were conducted in accordance with the Australian Code of Practice for the Care and Use of Animals for Scientific Purposes (National Health and Medical Research Council, 2013). Approval to proceed was granted by the Agricultural Research and Extension Animal Ethics Committee of the Department of Jobs, Precincts and Regions Animal Ethics Committee (DJPR, 475 Mickleham Road, Attwood, Victoria 3049, Australia), and the Tasmanian Department of Primary Industries, Parks, Water and Environment (DPIPWE Animal Biosecurity and Welfare Branch, 13 St Johns Avenue, New Town, Tasmania 7008, Australia). AEC project approval code 2017-05.

### 4.1. Animals and Datasets

A total of 298 Holstein-Friesian cows were used in this experiment. The calibration dataset (Dataset 1) was collected between August and September 2017 from 248 animals located at the Ellinbank Dairy Research Centre, Ellinbank, Victoria, Australia. An independent validation dataset (Dataset 2) was collected in September 2018, from 50 cows located on a commercial dairy farm in Smithton, Tasmania, Australia. All cows were clinically healthy, and had been calved for between 4 and 30 days at the time of sampling. Feeding systems on Australian dairy farms are diverse but can be classified into 5 main feeding systems [60]. Both farms operated under feeding system 2; grazed pasture plus moderate to high level concentrate feeding (>1.0 tonne of concentrate fed in the milking parlour per cow per year).

### 4.2. Blood Sample Collection and Reference Biomarker Measurements

A single serum sample was taken from each cow immediately after morning milking (approximately 07:00) according to the protocol described in Luke et al. [43]. Cows were fed their concentrate ration as soon as they entered the milking parlour, meaning that samples were collected approximately 10 min after grain feeding. 

An aliquot of each serum sample was shipped on ice to Regional Laboratory Services (Benalla, Victoria, Australia) for BHBA and NEFA analyses. Colorimetric assays were performed using a Kone 20 XT clinical chemistry analyser (Thermo Fisher Scientific, Waltham, MA, USA); an enzymatic kinetic assay for BHBA (McMurray et al., 1984) and enzymatic end point assay for NEFA (Randox Laboratories, Crumlin, UK). The uncertainty of measurement (at a 95% confidence level) was ±0.060 mmol/L at 0.85 mmol/L for BHBA, and ±0.031 mM at 1.45 mM for NEFA. A second aliquot was stored at −20 °C until processing for NMR spectroscopy.

### 4.3. Sample Preparation for NMR Spectroscopy

Details of the sample preparation and metabolite extraction protocols used in this study can be found in Luke et al. [43]. Briefly, 300 µL of serum was (1) mixed with 600 µL of methanol, (2) vortexed, (3) incubated at −20 °C for 20 min, and (4) centrifuged at 11,360× *g* at 21 °C for 30 min to pellet proteins. A 600 µL aliquot of the supernatant was then transferred to a clean 2 mL microcentrifuge tube, dried under vacuum at 21 °C overnight using a SpeedVac Savant SPD 2010 Concentrator (Thermo Fisher Scientific, Waltham, MA, USA) then reconstituted in a D_2_O phosphate buffer solution (100 mM K_2_HPO_4_) containing 0.25 mM DSS-d6 as an internal standard. A 550 µL aliquot of reconstituted extract was transferred to a 5 mm NMR tube for analysis.

### 4.4. ^1^H NMR Data Acquisition and Pre-Processing

One-dimensional proton spectra were acquired using a Bruker Ascend 700 MHz spectrometer equipped with cryoprobe and SampleJet automatic sample changer (Bruker Biospin, Rheinstetten, Germany). A Bruker noesypr1d pulse sequence was used over a −0.76–10.32 ppm spectral range with the following acquisition parameters; (1) a temperature of 298 K, (2) 256 scans after eight dummy scans (3) acquisition time per increment of 2.11 s, and (4) relaxation delay (D1) of 2.00 s. This resulted in 32,768 data points. A line broadening of 0.3 Hz was applied to all spectra prior to Fourier transformation. Spectra were manually phased, baseline corrected and referenced to the internal standard (DSS-d6) at δ 0.00 ppm using the Topspin v.3.6.1 software (Bruker Biospin, Rheinstetten, Germany).

Data pre-processing was performed in MatLab v.R20017b (Mathworks, Natick, MA, USA). Spectra were imported as a matrix of signal intensities using the ProMetab v.1.1 script [61]. Spectral pre-processing involved (1) deletion of the residual water peak region (δ 4.68–5.00 ppm), (2) spectral alignment using the correlation optimized warping algorithm [62], (3) normalization to total signal area (area = 1), (4) deletion of methanol (δ 3.32–3.36 ppm) and DSS-d6 (δ 0.4–0.60 ppm) peak regions, and the non-informative region beyond 9.00 ppm, (5) baseline correction using automatic weighted least squares and (6) mean centering.

### 4.5. Statistical Analysis

Statistical analysis of experimental metadata was performed in R v3.6.2 [63]. Differences between the 2 datasets were analysed using a paired *t*-test or a Wilcoxon signed-rank test depending on the normality of the data. 

Multivariate statistical analyses were performed using the PLS Toolbox v. 8.5.2 (Eigenvector Research Inc., Manson, WA, USA). Preliminary data analysis and outlier detection was performed using an unsupervised PCA. Examination of PC1 vs. PC2 scores plot showed 14 samples from Dataset 1 outside the 95% confidence level ellipse (Figure S1). These samples were individually examined, and a single spectrum with poor water suppression and baseline correction was removed from subsequent analyses. The influences of fixed effects (DIM, age and herd) on spectra were investigated using ANOVA simultaneous component analysis with 1000 permutations [64]. Untargeted metabolomic fingerprinting was done by regressing reference NEFA and BHBA concentrations against ^1^H NMR spectra using supervised OPLS regression. Variable importance of projection (VIP) scores for the first latent variable were used to identify the most statistically significant peaks in each model. Peaks of interest were identified using the Chenomx NMR suite software v.8.4 (Chenomx Inc., Edmonton, AB, Canada), comparison to the literature, 2D NMR analysis (COSY, gHMBC and gHSQC), and statistical total correlation spectroscopy [65].

OPLS models were constructed using data from Dataset 1. The robustness of models was assessed using (1) cross-validation using a venetian blind technique (10 sample splits with 1 sample per blind) and (2) external validation using data from Dataset 2. The prediction accuracy of OPLS models was assessed using the coefficient of determination (R^2^) and root mean square error (RMSE). Normalized RMSE (NRMSE) values, calculated as external validation RMSE divided by the interquartile interval of the observed data, were used to compare RMSE estimates for NEFA and BHBA predictions. Permutation testing (50 iterations and statistical significance determined using a Wilcoxon signed-rank test) was performed to ensure that models were not over-fitted. 

## 5. Conclusions

In this study we used an untargeted ^1^H NMR metabolomics approach to investigate the serum metabolic fingerprints of the two most common biomarkers of energy balance in dairy cows, BHBA and NEFA. Our results suggest that while BHBA and NEFA are indicative of similar metabolic states in early-lactation dairy cows, there are significant differences between the two biomarkers. Metabolites with common co-variances were intermediates of energy, phospholipid, and methyl donor metabolism. The most significant differences in the metabolomic fingerprints were related to acetate and creatine metabolism. We also identified several intermediate metabotypes which, when combined with genomic data, will enable further the investigation of the genetic architecture of metabolic health in early lactation dairy cows. 

## Figures and Tables

**Table 1:** Descriptive statistics of the datasets used in this experiment, including number of animals (N), stage of lactation defined as days in milk (DIM), age in years, and β-hydroxybutyrate (BHBA) and non-esterified fatty acid (NEFA) concentrations (mmol/L) in the serum obtained from clinically healthy dairy cows.

Variable	Dataset 1 (*N* = 248)			Dataset 2 (*N* = 50)			P^1^
	Min	Max	Mean (SD)	Min	Max	Mean (SD)	
DIM (days)	4	30	16.7 (6.0)	4	30	18.6 (7.3)	0.09
Age (years)	2	12	3.7 (2.0)	2	9	3.9 (1.8)	0.22
BHBA (mmol/L)	0.22	1.86	0.55 (0.21)	0.23	0.94	0.42 (0.17)	< 0.001
NEFA (mmol/L)	0.11	2.18	0.75 (0.32)	0.14	1.91	0.67 (0.36)	0.07

^1^ Statistical significance of the differences between Datasets 1 and 2 were determined using paired *t*-test for DIM, and a paired Wilcoxon signed-rank test for age, BHBA and NEFA.

## Supplementary Materials

The following are available online at https://www.mdpi.com/2218-1989/10/6/247/s1, Table S1: ^1^H NMR chemical shifts (δ) and multiplicity of metabolites in bovine serum run in deuterated water (D_2_O). Clearly observed resonances are indicated in bold text. s, singlet; d, doublet; dd, doublet of a doublet; m, multiplet; t, triplet. The right two columns show the direction of the relationship with serum β-hydroxybutyrate (BHBA) and non-esterified fatty acid (NEFA) concentrations determined by colorimetric assays, Table S2: Results of ANOVA-simultaneous component analysis (ASCA) of ^1^H NMR spectra of bovine serum (*N*= 298). Effect describes the relative influence of each variable (herd, age and days in milk (DIM)) on spectra. *p*-value is derived from permutation testing (1000 iterations), Figure S1: Results of PCA of ^1^H NMR spectra of serum obtained from 298 dairy cows in early lactation from the Ellinbank research farm (Dataset 1, *N* = 248) and a commercial dairy farm in Tasmania (Dataset 2, *N* = 50), Figure S2: VIP scores from OPLS regressions of ^1^H NMR spectra of serum obtained from 298 dairy cows in early lactation against (a) BHBA concentration and (b) NEFA concentration.
