# Supplementary material for: A Tale of Two Biomarkers: Untargeted 1H NMR Metabolomic Fingerprinting of BHBA and NEFA in Early Lactation Dairy Cows
Source: Metabolites. 2020 Jun 15;10(6):247. doi: 10.3390/metabo10060247 (PMC7345919; doi:10.3390/metabo10060247)
Supplement: Supplementary file 1 [file metabolites-10-00247-s001.pdf]

metabolites

MDPI

Supplementary Material

Table S1. ^{1}H NMR chemical shifts (δ) and multiplicity of metabolites in bovine serum run in deuterated water (D_2O). Clearly observed resonances are indicated in bold text**. s**, singlet; d, doublet; dd, doublet of a doublet; m, multiplet; t, triplet. The right two columns show the direction of the relationship with serum β-hydroxybutyrate (BHBA) and non-esterified fatty acid (NEFA) concentrations determined by colorimetric assays. * = tentative identification.

	Metabolite	Chemical shift (δ) and multiplicity	BHBA	NEFA
1	cholate*	**0.70 (m),** 0.91 (m), 0.96 (m), 1.43 (m), 1.87 (m), 2.10 (m), 2.22 (m), 3.65 (m), 4.06 (t)	-	↓
2	LDL/VLDL	**0.86 (m), 1.25 (m)**	↑	↑
3	leucine	**0.94 (d)**, **0.95 (d)**, 1.66 (m), 1.66 (m), 1.73 (m), 3.72 (m)	-	↑
4	isoleucine	**0.93 (t)**, **1.00 (d)**, 1.24 (m), 1.45 (m), 1.45 (m), 3.66 (d)	-	↓
5	valine	**0.98 (d)**, **1.03 (d)**, 2.26 (m), 3.60 (d)	↓	↓
6	β-hydroxybutyrate	**1.20 (d)**, **2.31 (m), 2.41 (m), 4.16 (m)**	↑	↑
7	lactate	**1.31 (d),** 4.31 (q)	↓	↓
8	alanine	**1.46 (d),** 3.77 (q)	↓	↓
9	acetate	**1.9 (s)**	↑	↓
10	N-acetyl glycoprotein	**2.03 (m)**	↑	↑
11	pyruvate	**2.46 (s),** 7.65 (s)	-	↓
12	citrate	**2.52 (d), 2.66 (d)**	-	-
13	creatine	**3.02 (s), 3.92 (s)**	↑	↓
14	phosphocreatine	**3.03 (s)**, **3.93 (s)**	-	-
15	dimethyl sulfone	**3.14 (s)**	↓	-
16	choline	**3.19 (s)**, 3.50 (m), 4.05 (m)	-	-
17	phosphocholine	**3.21 (s)**, 3.58 (t), 4.17 (m)	↑	↑
18	betaine	**3.25 (s)**, 3.89 (s)	↑	-
19	methanol	**3.34 (s)**		
20	glucose	**3.23 (dd)**, **3.40 (m), 3.46 (m), 3.52 (dd), 3.73 (m), 3.82 (m), 3.89 (dd), 4.63 (d)**, **5.22 (d)**	↓	↓
21	glycine	**3.50 (s)**	↑	↑
22	β-glucose	**4.63 (d)**	↓	↓
23	α-glucose	**5.22 (d)**	↓	↓
24	3-phenyllactate*	**2.87 (dd)**, 3.09 (dd), 4.26 (dd), **7.31 (m), 7.39 (m)**	-	-
25	hippurate	3.96 (d), **7.54 (m), 7.62 (m), 7.83 (dd)**	-	-
26	formate	**8.44 (s)**	-	-

metabolites

MDPI

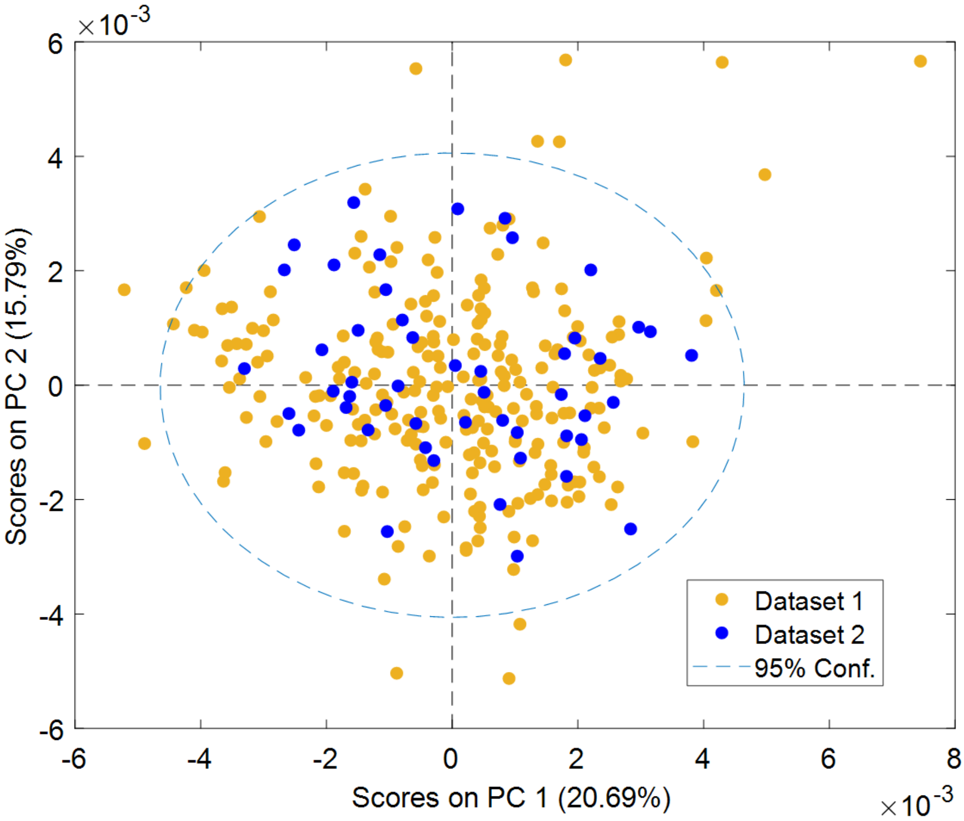

Figure S1. Results of PCA of ^{1}H NMR spectra of serum obtained from 298 dairy cows in early lactation from the Ellinbank research farm (Dataset 1, N = 248) and a commercial dairy farm in Tasmania (Dataset 2, N = 50).

Table S2. Results of ANOVA-simultaneous component analysis (ASCA) of ^{1}H NMR spectra of bovine serum (N = 298). Effect describes the relative influence of each variable (Herd, Age and days in milk (DIM)) on each spectra. P-value is derived from permutation testing (1000 iterations).

Variable	PCs	Effect	P-Value
Herd	1	0.47	0.33
Age	10	4.72	0.04
DIM	20	10.17	0.09
Residuals	-	86.06	-

metabolites

MDPI

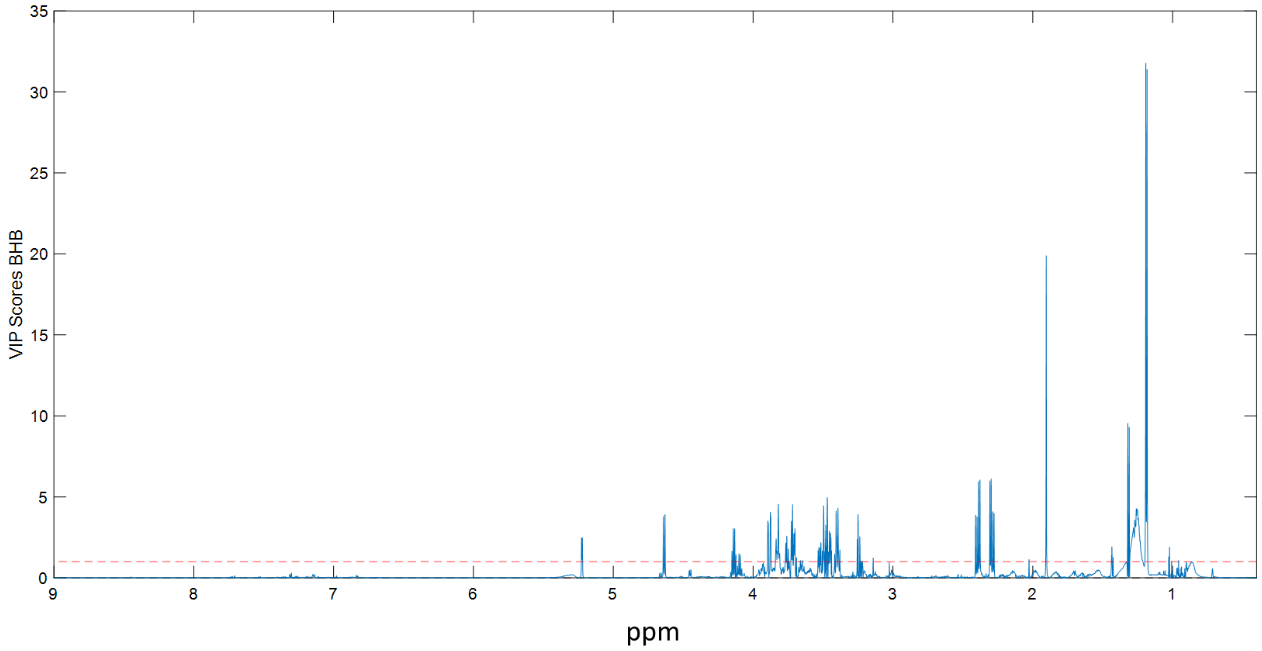

(a)

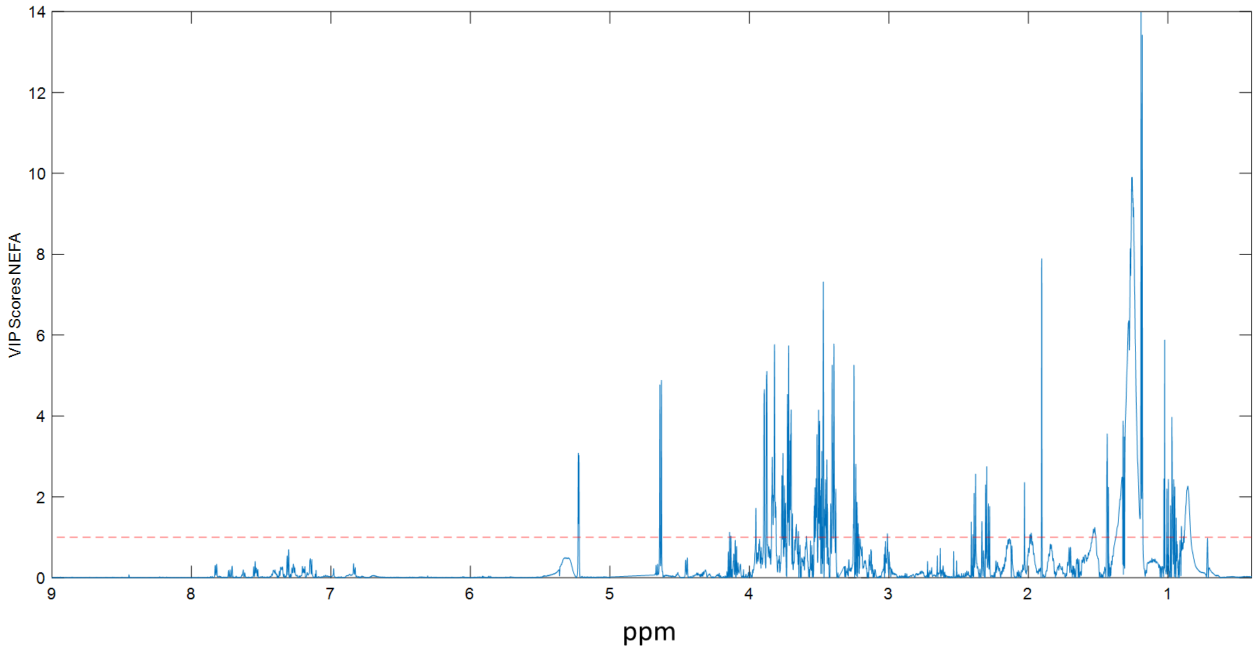

(b)

Figure S2. Variable importance of projection (VIP) scores derived from orthogonal partial least squares (OPLS) regression of ^{1}H NMR spectra of serum obtained from 298 dairy cows in early lactation, against (a) BHBA concentration and (b) NEFA concentration.
